# Differences in pregnancy outcomes in donor egg frozen embryo transfer (FET) cycles following preimplantation genetic screening (PGS): a single center retrospective study

**DOI:** 10.1007/s10815-016-0832-z

**Published:** 2016-11-16

**Authors:** Alison Coates, Brandon J. Bankowski, Allen Kung, Darren K. Griffin, Santiago Munne

**Affiliations:** 1 Oregon Reproductive Medicine, 808 SW 15th Ave., Portland, OR 97205 USA; 2 School of Biosciences, University of Kent, Canterbury, CT2 7NJ UK; 3 Reprogenetics, 3 Regent Street, Suite 301, Livingston, NJ 07039 USA

**Keywords:** Aneuploidy, Preimplantation diagnosis for aneuploidy (PGS), IVF, Donor egg

## Abstract

**Purpose:**

This study aims to test the hypothesis, in a single-center retrospective analysis, that live birth rates are significantly different when utilizing preimplantation genetic screening (PGS) compared to not utilizing PGS in frozen–thawed embryo transfers in our patients that use eggs from young, anonymous donors. The question therefore arises of whether PGS is an appropriate intervention for donor egg cycles.

**Methods:**

Live birth rates per cycle and live birth rates per embryo transferred after 398 frozen embryo transfer (FET) cycles were examined from patients who elected to have PGS compared to those who did not. Blastocysts derived from donor eggs underwent trophectoderm biopsy and were tested for aneuploidy using array comparative genomic hybridization (aCGH) or next-generation sequencing (NGS), then vitrified for future use (test) or were vitrified untested (control). Embryos were subsequently warmed and transferred into a recipient or gestational carrier uterus. Data was analyzed separately for single embryo transfer (SET), double embryo transfer (DET), and for own recipient uterus and gestational carrier (GC) uterus recipients.

**Results:**

Rates of implantation of embryos leading to a live birth were significantly higher in the PGS groups transferring two embryos (DET) compared to the no PGS group (GC, 72 vs. 56 %; own uterus, 60 vs. 36 %). The live birth implantation rate in the own uterus group for SET was higher in the PGS group compared to the control (58 vs. 36 %), and this almost reached significance but the live birth implantation rate for the SET GC group remained the same for both tested and untested embryos. Live births per cycle were nominally higher in the PGS GC DET and own uterus SET and DET groups compared to the non-PGS embryo transfers. These differences almost reached significance. The live birth rate per cycle in the SET GC group was almost identical.

**Conclusions:**

Significant differences were noted only for DET; however, benefits need to be balanced against risks associated with multiple pregnancies. Results observed for SET need to be confirmed on larger series and with randomized cohorts.

## Introduction

Approximately 12 % of all in vitro fertilization (IVF) cycles in the US are completed using donor eggs from young anonymous egg donors, which equated to over 20,000 cycles in 2013 [1]. Reasons for using donor eggs from young women rather than autologous eggs during cycles of IVF are varied. They include advanced maternal age, premature ovarian failure, diminished ovarian reserve, multiple failed IVF cycles using own eggs, oophorectomy, same-sex male couples, maternal single gene defects, and cancer treatment in the female patient [1].

As aneuploidy frequency in human preimplantation embryos increases with maternal age [2, 3], implantation and live birth rates decrease [1] because most aneuploid embryos either fail to implant [4] or miscarry and, rarely, are compatible with life [5, 6]. National IVF live birth rates for embryo transfers (ET) using fresh embryos compiled by the Centre for Disease Control in 2013 from patients younger than 35 were 48 % per ET compared to patients aged 41–42 with a live birth rate of only 16 % per ET [1]. While fresh embryo transfers using donor eggs have resulted in high live birth rates without PGS (live birth rate 77 %/ET 2012 SART published Oregon Reproductive Medicine (ORM) data [7]), frozen ET live birth rates in this group have been lower. Improvements in freezing techniques over the last 10 years have led to better survival of embryos and therefore higher pregnancy rates. National US data [1] comparing fresh versus frozen ETs in the donor egg recipient group showed that in 2003, fresh live birth rate/ET was 51 % compared to frozen ET at 30 %. In 2013, the national fresh live birth rate/ET rate was 56 % compared to 40.5 % with frozen ET. The gap between fresh and frozen embryo transfers has thus become less over this time period from a 21 % difference in 2003 to a 14.5 % difference in 2013, partly attributable to improved freezing methods.

PGS of blastocysts before transfer, it has been suggested, can reduce the maternal age effect on implantation [8], significantly increase live birth rates, and reduce miscarriage risk in IVF cycles when using autologous oocytes [9–16]. This area is perhaps among the most contentious in reproductive medicine however with significant proponents and opponents on both sides [17, 18]. Meta-analyses and randomized controlled clinical trials have demonstrated the benefits of PGS for high-risk groups (e.g., advanced maternal age, recurrent implantation failure); however, analyses and conclusions still attract criticism [19, 20]. At the time of writing, the community awaits the results of the Illumina STAR trial for PGS (trial registration number NCT02268786).

To date, most attention has been on the high-risk referral categories; however, many IVF/PGD practitioners have proposed that all IVF embryos should be screened for aneuploidy prior to transfer, including those from younger patients [21]. Although the percentage of aneuploid blastocysts from young donors is low compared to older patients (25 % for donor eggs vs. 60 % for 41–43-year-old women, ORM unpublished data), it is nonetheless a concern since most aneuploidies arise from maternal meiotic errors in the eggs of younger women [22]. Sills et al. determined, using SNP technology, the parental origin of aneuploidy using donor eggs [23], finding that 88 % of all the aneuploidies were attributable to maternal errors. Despite this, egg donor cycles have historically been the least likely group to be offered this screening.

To the best of our knowledge, there has only been one study examining the use of PGS in frozen–thawed donor egg derived embryos [24]. Their conclusion was that, although their dataset was small, there was an increase (not significant) in implantation rates and pregnancy rates in the PGS group compared to the non-tested group. In the absence of any statistical significance, however, results remain unconvincing. A larger study is thus needed, as is stratifying patients into “own” and “gestational carrier” uterus as well as double versus single embryo transfers. In this study, we thus tested the hypothesis that ongoing pregnancy and implantation rates in frozen embryo transfer (FET) cycles are significantly different in FET cycles utilizing PGS compared to FET cycles not utilizing PGS in a single center.

## Materials and methods

### Ethical statement

All patients were consented for all procedures as part of routine care. Egg donors were all anonymous. Institutional review board approval was obtained for review of patient charts and laboratory data for this study. The University of Kent Research and Ethics Committee also approved this study.

### Overview

Blastocysts resulting from donor egg IVF cycles at the Oregon Reproductive Medicine (ORM) clinic between January 2013 and December 2015 either elected to be tested for aneuploidy by trophectoderm (TE) biopsy followed by PGS using aCGH [25] or NGS [26] before cryopreservation; otherwise, their embryos were cryopreserved without testing depending on patient preference. All blastocysts subsequently transferred in this retrospective observational study were at the day 5 stage when biopsied and frozen.

### Recipient population and study design

Patients using donor eggs from Caucasian donors to create embryos for their IVF cycle were included in this retrospective analysis. The patients were divided into two main groups: those using their own uterus and those using a gestational carrier (GC) uterus for the embryo transfer. These groups were further divided into those transferring a single embryo (SET) and those transferring two embryos (double embryo transfer, DET). The groups were then finally divided into those transferring apparently euploid embryos screened by PGS (study group) and those transferring non-screened embryos (control group).

### Donor stimulation, embryo culture, and blastocyst biopsy

Controlled ovarian stimulation protocols for these IVF cycles were carried out as previously described [27]. On completion of the retrieval procedure, oocytes were placed in Quinns Advantage Fertilization Medium (Origio, USA) supplemented with 5 % human serum albumin (HSA) (Irvine Scientific, USA) under oil (Ovoil, Vitrolife, USA), and ICSI or standard insemination performed 4 h after retrieval [28]. Once all eggs had been either inseminated or injected, they were returned to the incubator for overnight culture. All embryos were moved to Quinns Advantage Cleavage Medium (Sage, Origio, USA) supplemented with 10 % HSA (Irvine Scientific) from days 1 to 3 and subsequently moved to Quinns Advantage Blastocyst Medium (Sage, Origio, USA) supplemented with 10 % HSA from days 3 to 6.

All embryos to be biopsied were hatched on day 3 post-retrieval using a Hamilton Thorne Zilos™ laser with an 800-μm pulse then allowed to develop in culture media until day 5 or 6 of development. Embryos were considered suitable for biopsy on day 5 when at least 10 % of the TE was protruding from the breach in the zona pellucida made on day 3. All embryos that had not fully expanded by day 5 were cultured until day 6 and then biopsied. Embryos were only biopsied if there was a visible inner cell mass (ICM) and multi-celled TE protruding from the zona pellucida. Embryos that grew to an expanded blastocyst stage had three to eight TE cells excised using a Hamilton Thorne Zilos™ laser with an 800-μm pulse. Biopsied embryos were then vitrified using Irvine vitrification media with DMSO (Irvine Scientific, CA) on Cryotops (Kitazawa, Japan) and stored for future use. Biopsied cells were sent to the Reprogenetics Laboratory for analysis using NGS or aCGH.

### DNA analysis

The biopsied cells were whole genome amplified (WGA) using the Sureplex DNA Amplification System (Bluegnome) according to the manufacturer’s protocol. The WGA products were then processed for aneuploidy analysis by NGS or aCGH. Testing by aCGH was processed using Bluegnome 24sure V3 protocol (Illumina, Inc.) according to the manufacturer’s protocol. WGA products were fluorescently labeled with Cy3 and Cy5 dyes and random primers and subsequently were prepared to be hybridized to 24sure V3 array slides. Aneuploidy data analysis was performed using BlueFuse Multi Software. Testing by NGS was processed using Ion Torrent PGM (Ion Torrent) technology. Libraries were prepared by fragmenting WGA products with DNA concentrations of 100 ng using Ion Xpress Plus Fragment Library Kit (Life Technologies). Library fragments were selected at 200 bp using E-Gel SizeSelect Gels (Life Technologies) and then were normalized to 100 pM using an Ion Library Equalizer kit (Life Technologies). Libraries were subsequently pooled together into a mastermix and clonal amplified on the Ion One Touch 2 system. The template was then loaded into a 316 V2 chip (Life Technologies) and sequenced at 200 bp. Aneuploidy data analysis was performed using Ion Reporter software, using Low-Coverage Whole-Gnome workflow.

### Uterine preparation and blastocyst warming protocol for embryo transfer

Embryo transfers of previously vitrified blastocyst stage embryos were carried out in a medicated uterine preparation cycle as described previously [29]. Embryos were warmed using Irvine warming kits (Irvine Scientific, Irvine, CA) on the morning of the scheduled embryo transfer and allowed to re-expand in equilibrated drops of Embryoglue™ (Vitrolife) under Ovoil™ (Vitrolife) until the time of transfer. Embryo transfer was carried out using a Wallace Sureview Embryo transfer catheter (CooperSurgical) under ultrasound guidance. Patients rested for 45 min post-transfer.

### Measured outcomes and statistical analysis

Measured outcomes were live birth rate per cycle and live birth implantation rate (number of babies born per embryo transferred). Any statistical differences in each of these parameters for each group were established using Fisher’s exact test and Mann-Whitney test on ranks where appropriate. Statistical significance was determined at *p* <0.05 at the 95 % confidence level. The tested and non-tested groups had near identical average egg donor age, and the number of embryos biopsied in each group was not statistically significantly different (Table 1).

**Table 1:** Comparison of donor age and numbers of embryos vitrified in test and control groups

	Own uterus		Gestational carrier uterus		Total	
	PGS	No PGS	PGS	No PGS	PGS	No PGS
Average donor age (range)	25.6 (20–33)	25.4 (19–32)	25 (21–30)	25.4 (21–30)	25.2 (20–33)	25.4 (19–32)
*p* value	>0.5 not significant		>0.5 not significant		>0.5 not significant	
Average embryos vitrified	6.9	6.4	7.9	7.6	7.5	7.0
*p* value	>0.5 not significant		>0.5 not significant		>0.5 not significant	

Results demonstrate highly similar (i.e., not significantly different) numbers

During the study period, personnel in the laboratory all remained constant, and there were no major changes to protocols during the time period of the study.

## Results

A total of 398 thaw cycles were included in the analysis. One thaw cycle resulted in no transfer due to failure to survive of the one available embryo. A total of 397 frozen ETs were performed using blastocyst stage embryos generated from eggs from anonymous donors. All embryos were classed as of good morphological appearance (AA grade, Gardner scale) [30] prior to cryopreservation. In the test (PGS) group, 435 known euploid embryos were transferred in 294 frozen ET cycles. In the control (no PGS) group, 103 embryos of unknown ploidy status were transferred in 162 frozen ET cycles.

There was no difference in survival of embryos post-thaw in both the PGS and control groups (96 % in the PGS group and 97 % in the control group, *p* = 1.0 NS). No patients using donor eggs during the time period of the study had a cycle with all aneuploid embryos.

Results are summarized in Table 2. Live birth implantation rates (number of babies born per embryo transferred) were significantly higher in the PGS group in both the GC and own uterus groups, but only after double embryo transfer (DET) (72 vs. 56 % GC, 60 vs. 36 % own uterus; *p* values = 0.03 and 0.005, respectively). The live birth rates per transfer cycle in the DET groups were nominally higher in the PGS group compared to control group but not significantly so (87 vs. 77 % GC, 76 vs. 55 % own uterus; *p* values = 0.2 and 0.08, respectively). Live birth implantation rates and live births per cycle in the GC SET group were the same in the PGS and own uterus group, but the control group was small including only 20 cycles. Live birth implantation rates (58 vs. 36 %, *p* = 0.09) and live births per cycle (58 vs. 36 %, *p* = 0.09) were nominally higher with PGS versus control in the SET own uterus group but not significantly.

**Table 2:** Outcome measures: live births per cycle and live birth implantation rate (number of babies born per embryo transferred) in test (PGS) versus control (no PGS) groups

	Gestational carrier uterus				Own recipient uterus			
	SET		DET		SET		DET	
	PGS (test)	No PGS (control)	PGS (test)	No PGS (control)	PGS (test)	No PGS (control)	PGS (test)	No PGS (control)
Number of thaw cycles	95	20	100	26	58	25	41	33
Number of FETs	95	19	100	26	58	25	41	33
Number of embryos transferred	95	19	200	52	58	25	82	66
Number of ongoing pregnancies** (%/FET)	66 (70 %)	13 (68 %)	93 (93 %)	22 (84 %)	37 (64 %)	10 (40 %)	34 (83 %)	20 (61 %)
*p* value	1.0		0.2		0.06		0.04	
Number of live births (%/thaw cycle)	58 (61 %)	12 (60 %)	87 (87 %)	20 (77 %)	34 (58 %)	9 (36 %)	31 (76 %)	18 (55 %)
*p* value	1.0		0.2		0.09		0.08 not quite significant	
Number of babies born (LB rate/embryo transferred)	58 (61 %)	12 (63 %)	144 (72 %)	29 (56 %)	34 (58 %)	9 (36 %)	49 (60 %)	24 (36 %)
*p* value	1.0		0.03		0.09		0.005	
Twinning (rate/live birth)	0	0	58 (67 %)	9 (45 %)	0	0	18 (58 %)	6 (33 %)
*p* value			0.07				0.1 NS	

Finally, live birth twinning rates were nominally higher following PGS for DET cases (67 vs. 45 % for GC uterus, *p* = 0.07 and 58 vs. 33 % for recipient own uterus, *p* = 0.1 in test and control groups, respectively) compared to zero monozygotic twinning following SET in either the test or control group.

Table 3 illustrates that 25 % of all donor egg derived trophectoderm samples tested were aneuploid, 19 % with a single aneuploidy and 5.7 % with more than one chromosome involved. Table 3 breaks down the errors by chromosome, with 4.6 % of embryos resulting in a chromosomal aneuploidy commonly associated with miscarriage or abnormal offspring (i.e., trisomies 13, 15, 16, 18, 21, 22, XY, and monosomy X). aCGH and NGS methodologies resulted in similar overall aneuploidy rates per embryo (24 % for aCGH vs. 26 % for NGS, respectively, *p* > 0.05 not significant). Monosomies and trisomies occurred at similar frequencies (overall and per chromosome) with chromosome 16 and the sex chromosomes most commonly aneuploid.

**Table 3:** Results of aneuploidy rates and specific chromosomes affected for 3393 donor egg derived blastocysts biopsied between 2012 and 2015

Number of embryos biopsied	3393	
Total with aneuploidy (% per embryo)	835 (25 %)	
Number with complex aneuploidy (>1 chromosome aneuploid) (% per embryo)	193 (5.7 %)	
Number with single aneuploidy (% per embryo)	642 (19 %)	
Single aneuploidy only:		
Chromosome:	Number of embryos with monosomy	Number of embryos with trisomy
1	18	8
2	19	10
3	7	9
4	17	8
5	13	14
6	16	11
7	21	6
8	17	12
9	10	17
10	11	9
11	16	8
12	5	11
13	19	6 (0.2 %)^a^
14	13	6
15	22	16 (0.5 %)^a^
16	38	37 (1.1 %)^a^
17	6	3
18	9	4 (0.1 %)^a^
19	11	10
20	4	9
21	13	11 (0.3 %)^a^
22	24	15 (0.4 %)^a^
Sex chromosomes	35 (XO) (1 %)^a^	32 (0.9 %)^a^
Total number of embryos with most common aneuploidies associated with implantation and subsequent miscarriage or abnormal live birth^a^ (% per embryo)	156 (4.6 %)	

^a^Indicates the 4.6 % of embryos representing the most common trisomies associated with spontaneous abortion and live birth

## Discussion

Donor egg IVF cycles have many variants. If the female donor egg recipient is able to carry the pregnancy herself, the embryos are transferred to her uterus. If, however, she has an abnormal uterus or no uterus at all, the recipient may engage a gestational carrier to gestate the pregnancy. Same-sex male couples or single men can fertilize donor eggs with their own sperm and transfer resulting embryos to a gestational carrier. While the live birth rate of fresh donor egg embryos is high overall, if the cases which used a gestational carrier are separated out from the recipients who used their own uterus, we find an even higher pregnancy rate in the donor egg gestational carrier patients than when using a donor egg recipient uterus from an infertile patient. This even higher pregnancy rate when using a gestational carrier who has successfully previously carried a baby to term could be attributable to the elimination of unknown uterine factors that may contribute to failure to achieve a successful pregnancy in patients who have been infertile for long periods of time.

In this study, patients elected whether or not to test their embryos by PGS before cryopreservation. The people who elected not to test had the same prognosis as the patients who did elect to test, i.e., all patients presenting in our anonymous egg donor program had a high chance of conceiving and had access to the same pool of young egg donors. Patients committed to the testing process before the cycle started and patients did not change their mind depending on how many blastocysts developed. The reasons patients elected not to test were financial, uncertainty about the biopsy process, and a wish to leave the choosing of embryos up to chance.

Egg donor cycles have historically culminated in a transfer of fresh, untested embryos to a recipient or gestational carrier uterus or a freeze-all cycle for future use. With an average of seven good quality blastocysts produced each donor egg cycle, even if a fresh transfer is undertaken, there are usually surplus embryos remaining to be cryopreserved for future use [31]. Often an egg donor cycle cannot be synchronized for a fresh embryo transfer with a recipient or gestational carrier uterus for social or medical reasons and cryopreservation of the whole embryo cohort (“freeze all”) becomes necessary [32, 33]. The findings of this study indicate that there are possible benefits in implantation and ongoing pregnancy rates through the use of PGS in FET donor egg cycles. The question of whether FET cycles derived from donor eggs need to include PGS in future should thus now be subject of larger and ultimately randomized studies.

Single-center retrospective cohort studies such as this one provide supportive evidence to justify future prospective cohort studies, multi-center meta-analyses, and ultimately randomized controlled clinical trials (RCTs). For evidence-based reproductive medicine, each has their value. RCTs remain the gold standard, but in a field where individual operator skills can have such a profound effect on IVF outcome, larger studies can mask effect of good (or bad) practice of individual groups or centers. In this context, smaller single-center trials have considerable value and may point to genuine efficacy of clinical interventions. On the other hand, the absence of randomization leaves an open question of whether any significant differences observed represent selection bias, inadvertent or otherwise. Moreover, different sizes of test and control groups (in this case, control groups are much smaller than test groups) mean that statistical analyses need to be viewed with a degree of caution. RCTs are however expensive, time consuming, and cannot be justified without sufficient published datasets from retrospective analyses. In our opinion, the current study justifies further work in this area, particularly with the increased interest in IVF and PGS.

With all the above caveats in mind, the current study is nonetheless the first to show significant differences as a result of the use of PGS in patients who elected to have it as part of their FET donor egg treatment cycle. Apparent improvements were seen after double embryo transfer (DET), raising the question of whether differences are only likely to be seen in this context. Equally, as the IVF world in most countries moves more in the direction of single embryo transfers (SET) [34–36], attention will inevitably turn to means through which the chances of implantation of that single embryo can be maximized. In our SET GC group, there was no difference in live births per cycle nor live birth implantation rates per embryo between the PGS and no PGS embryos, and in the SET group using a recipient uterus, there was a nominal increase in those parameters when using PGS but not significantly. Taking gestational carrier and own uterus cycles together, all measures favored the PGS cycles, but in the absence of statistical significance, the question remains open about whether this represented a genuine difference. The DET results suggest that, had sufficient numbers been analyzed, the numbers might have reached statistical significance for SET also; however, this needs to be confirmed by further studies. Elective single embryo transfer (SET) to reduce multiple pregnancy rates in IVF cycles is a primary goal of the reproductive medicine community [35]. SETs, however, result in lower pregnancy rates per transfer and higher rates of complete pregnancy loss (post-positive pregnancy test) compared to double embryo transfer (DET). The benefit of transferring a single embryo is the reduction in the number of twin pregnancies because of the obstetric complications associated with multiple births. In future studies, therefore, we would look closely to ask whether the nominal differences seen for SET in this study reach statistical significance in larger data sets.

Without randomization, the question also arises about whether the statistical differences that were observed reflect patient cohorts that inherently had differing aneuploidy rates. Of course, we cannot completely rule this out. In these particular patient groups, however, patients had near identical egg donor ages, the best known correlate for different aneuploidy rates [2, 3, 8]. Equally, the couples who elected not to test had the same prognosis as the patients who did elect to have PGS, i.e., all patients presenting in our anonymous egg donor program had a high chance of conceiving and had access to the same pool of young egg donors. Patients committed to the testing process before the cycle started and patients did not change their mind depending on how many blastocysts developed. The reasons patients elected not to test were financial, uncertainty about the biopsy process, and a wish to leave the choosing of embryos up to chance. Whether these are confounding factors that might predispose to increased aneuploidy or reduced pregnancy is questionable. In a study of 23 US clinics sending biopsies to a single reference laboratory (Reprogenetics), Munne et al. showed that aneuploidy rates in donor egg embryos vary between IVF clinics [37]. The percent of aneuploid donor egg embryos per clinic ranged from 20 to 58 % with an average rate of 35 %. One possible explanation for this is geographical variation in aneuploidy rates; however, there is scant data supporting this hypothesis. A more likely explanation is subtle differences in ovarian stimulation and laboratory protocols might lead to marked differences in aneuploidy levels between centers. Such questions are beyond the scope of this study and are not entirely relevant in this case as this is a single-center study in which personnel did not change and identical standard operating practices were performed. Indeed, in our own experience, donor egg aneuploidy rates are lower than the average shown in the Munne study [26]. Nonetheless, we cannot rule out inadvertent, subtle differences between treatments of test versus control groups, however unlikely we believe it to be. It is perhaps not unreasonable to suggest, however, that the centers with higher aneuploidy rates in embryos derived from donor eggs might be the ones that benefit most from PGS, should it ultimately prove effective for this application.

Donor egg pregnancies, although at lower risk for miscarriage or offspring with chromosomal defects than pregnancies from patients of advanced maternal age, are still at risk from adverse pregnancy outcomes. The most common single aneuploidies associated with miscarriage or congenitally affected live births are trisomies 13, 15, 16, 18, 21, 22, XY, and monosomy X [38, 39]. In this retrospective analysis, 4.6 % of blastocysts fall into this category (Table 3). Given that monosomies (other than monosomy X) are rarely, if ever, seen among spontaneous abortions, we might expect these and other trisomies (e.g., trisomies 1 and 19) not to reach the stage of a clinically recognized pregnancy, perhaps through failed implantation. Others, however, such as the more common trisomies above, remain a significant concern for donor egg recipients when using unscreened embryos for transfer, especially since prenatal diagnosis is not routinely recommended for this group. Other features also noteworthy from Table 3 include monosomies and trisomies that occur with equal frequency indicating that embryos are equally likely to survive to the blastocyst stage whether they arise from a nullisomic or disomic gamete. Moreover, the most common aneuploidies seen in spontaneous abortions (monosomy X and trisomy 16) [40] are the most common in this dataset, suggesting that some patterns of chromosome-specific rates of aneuploidies are laid down before implantation.

PGS costs around $4500 (including biopsy procedure plus testing process) per cycle in addition to the IVF cycle costs. The cost including medication of one FET cycle is also around $4500. As 45 % of non-PGS embryos fail to implant and result in a pregnancy compared to 22 % of PGS embryos, it is more likely that a second or maybe third frozen embryo transfer may be needed to achieve a successful outcome when using untested embryos, therefore offsetting the initial cost of embryo screening.

Aneuploidy is a condition that affects all age groups, even women in their twenties [3]. While it has been suggested that PGS is a valuable tool for improving outcome in patients of advanced maternal age [8]; in the current study, we provide preliminary evidence that the application of PGS may improve IVF outcomes using younger oocytes from an egg donation cycle. This research provides sufficient evidence for increased research in this area, ultimately leading to prospective randomized clinical trials to address this issue further.

## References

[1] SART. Published IVF data 2013.

[2] Munne S, Chen S, Colls P, Garrisi J, Zheng X, Cekleniak N (2007). Maternal age, morphology, development and chromosome abnormalities in over 6000 cleavage-stage embryos. Reprod BioMed Online.

[3] Franasiak JM, Forman EJ, Hong KH, Werner MD, Upham KM, Treff NR (2014). The nature of aneuploidy with increasing age of the female partner: a review of 15,169 consecutive trophectoderm biopsies evaluated with comprehensive chromosomal screening. Fertil Steril.

[4] Munne S, Bahce M, Sandalinas M, Escudero T, Marquez C, Velilla E (2004). Differences in chromosome susceptibility to aneuploidy and survival to first trimester. Reprod BioMed Online.

[5] Werner M, Reh A, Grifo J, Perle MA (2012). Characteristics of chromosomal abnormalities diagnosed after spontaneous abortions in an infertile population. J Assist Reprod Genet.

[6] Kim JW, Lee WS, Yoon TK, Seok HH, Cho JH, Kim YS (2010). Chromosomal abnormalities in spontaneous abortion after assisted reproductive treatment. BMC Med Genet.

[7] SART. ORM live birth rates 2012.

[8] Harton GL, Munne S, Surrey M, Grifo J, Kaplan B, McCulloh DH (2013). Diminished effect of maternal age on implantation after preimplantation genetic diagnosis with array comparative genomic hybridization. Fertil Steril.

[9] Schoolcraft WB, Fragouli E, Stevens J, Munne S, Katz-Jaffe MG, Wells D (2010). Clinical application of comprehensive chromosomal screening at the blastocyst stage. Fertil Steril.

[10] Schoolcraft WB, Katz-Jaffe MG (2013). Comprehensive chromosome screening of trophectoderm with vitrification facilitates elective single-embryo transfer for infertile women with advanced maternal age. Fertil Steril.

[11] Hodes-Wertz B, Grifo J, Ghadir S, Kaplan B, Laskin CA, Glassner M (2012). Idiopathic recurrent miscarriage is caused mostly by aneuploid embryos. Fertil Steril.

[12] Scott Jr RT, Upham KM, Forman EJ, Hong KH, Scott KL, Taylor D, et al. Blastocyst biopsy with comprehensive chromosome screening and fresh embryo transfer significantly increases in vitro fertilization implantation and delivery rates: a randomized controlled trial. Fertil Steril. 2013;100(3):697–703. 10.1016/j.fertnstert.2013.04.035 23731996

[13] Yang Z, Liu J, Collins GS, Salem SA, Liu X, Lyle SS (2012). Selection of single blastocysts for fresh transfer via standard morphology assessment alone and with array CGH for good prognosis IVF patients: results from a randomized pilot study. Mol Cytogenet.

[14] Forman EJ, Hong KH, Treff NR, Scott RT (2012). Comprehensive chromosome screening and embryo selection: moving toward single euploid blastocyst transfer. Semin Reprod Med.

[15] Dahdouh EM, Balayla J, Audibert F, Wilson RD, Audibert F, Brock JA (2015). Technical update: preimplantation genetic diagnosis and screening. J Obstet Gynaecol Can JOGC J d’obstetrique et gynecologie du Can JOGC.

[16] Lee E, Illingworth P, Wilton L, Chambers GM (2015). The clinical effectiveness of preimplantation genetic diagnosis for aneuploidy in all 24 chromosomes (PGS): systematic review. Hum Reprod.

[17] Chen M, Wei S, Hu J, Quan S (2015). Can comprehensive chromosome screening technology improve IVF/ICSI outcomes? A meta-analysis. PLoS ONE.

[18] Verpoest W, Haentjens P, De Rycke M, Staessen C, Sermon K, Bonduelle M (2009). Cumulative reproductive outcome after preimplantation genetic diagnosis: a report on 1498 couples. Hum Reprod.

[19] Sahin L, Bozkurt M, Sahin H, Gurel A, Yumru AE (2014). Is preimplantation genetic diagnosis the ideal embryo selection method in aneuploidy screening?. Kaohsiung J Med Sci.

[20] Dahdouh EM, Balayla J, Garcia-Velasco JA (2015). Comprehensive chromosome screening improves embryo selection: a meta-analysis. Fertil Steril.

[21] Munne S, Ary J, Zouves C, Escudero T, Barnes F, Cinioglu C (2006). Wide range of chromosome abnormalities in the embryos of young egg donors. Reprod BioMed Online.

[22] Ata B, Kaplan B, Danzer H, Glassner M, Opsahl M, Tan SL (2012). Array CGH analysis shows that aneuploidy is not related to the number of embryos generated. Reprod BioMed Online.

[23] Sills ES, Li X, Frederick JL, Khoury CD, Potter DA (2014). Determining parental origin of embryo aneuploidy: analysis of genetic error observed in 305 embryos derived from anonymous donor oocyte IVF cycles. Mol Cytogenet.

[24] Haddad G, Deng M, Wang CT, Witz C, Williams D, Griffith J, et al. Assessment of aneuploidy formation in human blastocysts resulting from donated eggs and the necessity of the embryos for aneuploidy screening. J Assist Reprod Genet. 2015;32(6):999–1006. 10.1007/s10815-015-0492-4 PMC4491073 25956263

[25] Gutierrez-Mateo C, Colls P, Sanchez-Garcia J, Escudero T, Prates R, Ketterson K (2011). Validation of microarray comparative genomic hybridization for comprehensive chromosome analysis of embryos. Fertil Steril.

[26] Wells D, Kaur K, Grifo J, Glassner M, Taylor JC, Fragouli E (2014). Clinical utilisation of a rapid low-pass whole genome sequencing technique for the diagnosis of aneuploidy in human embryos prior to implantation. J Med Genet.

[27] Schoolcraft WB, Gardner DK (2000). Blastocyst culture and transfer increases the efficiency of oocyte donation. Fertil Steril.

[28] Palermo G, Joris H, Devroey P, Van Steirteghem AC (1992). Pregnancies after intracytoplasmic injection of single spermatozoon into an oocyte. Lancet.

[29] Roque M, Valle M, Guimaraes F, Sampaio M, Geber S (2015). Freeze-all policy: fresh vs. frozen-thawed embryo transfer. Fertil Steril.

[30] GGardner D, Schoolcraft W. In Vitro Culture of human Blastocysts. Jansen R, Mortimer D (Eds). *Toward Reproductive certainty: Fertility, Genetics and Beyond*. Carnforth, UK. Parthenon Publishing; 1999. P. 378–88.

[31] Doherty LF, Martin JR, Kayisli U, Sakkas D, Patrizio P (2014). Fresh transfer outcome predicts the success of a subsequent frozen transfer utilizing blastocysts of the same cohort. Reprod BioMed Online.

[32] McLernon DJ, Maheshwari A, Lee AJ, Bhattacharya S (2016). Cumulative live birth rates after one or more complete cycles of IVF: a population-based study of linked cycle data from 178 898 women. Hum Reprod.

[33] Zenke U, Chetkowski RJ (2004). Transfer and uterine factors are the major recipient-related determinants of success with donor eggs. Fertil Steril.

[34] Styer AK, Luke B, Vitek W, Christianson MS, Baker VL, Christy AY, et al. Factors associated with the use of elective single-embryo transfer and pregnancy outcomes in the United States, 2004–2012. Fertil Steril. 2016;104(3):e102. 10.1016/j.fertnstert.2016.02.034 PMC5451272 26997248

[35] Forman EJ, Hong KH, Franasiak JM, Scott RT (2014). Obstetrical and neonatal outcomes from the BEST Trial: single embryo transfer with aneuploidy screening improves outcomes after in vitro fertilization without compromising delivery rates. Am J Obstet Gynecol.

[36] Ubaldi FM, Capalbo A, Colamaria S, Ferrero S, Maggiulli R, Vajta G (2015). Reduction of multiple pregnancies in the advanced maternal age population after implementation of an elective single embryo transfer policy coupled with enhanced embryo selection: pre- and post-intervention study. Hum Reprod.

[37] Munne S, Alikani M, Barritt J, Hesla J, Kaplan B, Alper M, McCulloh D. Egg donor aneuploidy rates significantly differ between fertility centers. ASRM abstract submitted. 2015.

[38] Jenderny J (2014). Chromosome aberrations in a large series of spontaneous miscarriages in the German population and review of the literature. Mol Cytogenet.

[39] Hook EB, Cross PK, Schreinemachers DM (1983). Chromosomal abnormality rates at amniocentesis and in live-born infants. JAMA J Am Med Assoc.

[40] Shen J, Wu W, Gao C, Ochin H, Qu D, Xie J (2016). Chromosomal copy number analysis on chorionic villus samples from early spontaneous miscarriages by high throughput genetic technology. Mol Cytogenet.

